# Two-Year Follow-Up Study of Patients with Neovascular Age-Related Macular Degeneration Undergoing Anti-VEGF Treatment during the COVID-19 Pandemic

**DOI:** 10.3390/jcm13030867

**Published:** 2024-02-01

**Authors:** Jae-Gon Kim, Yu Cheol Kim, Kyung Tae Kang

**Affiliations:** 1 Department of Ophthalmology, Keimyung University School of Medicine, Keimyung University Dongsan Hospital, Daegu 42601, Republic of Korea; jgkim9418@kaist.ac.kr (J.-G.K.); eyedr@dsmc.or.kr (Y.C.K.); 2 Graduate School of Medical Science and Engineering, Korea Advanced Institute of Science and Technology, Daejeon 34141, Republic of Korea

**Keywords:** vascular endothelial growth factors, COVID-19, macular degeneration

## Abstract

Background: regular intravitreal anti-vascular endothelial growth factor (VEGF) treatment is crucial for patients with neovascular age-related macular degeneration (nAMD), and delayed treatment can exacerbate disease progression. Methods: we compared the outcomes of on-time versus delayed intravitreal anti-VEGF treatment for patients with nAMD. This study was conducted during the coronavirus disease 2019 (COVID-19) pandemic with a 2-year follow-up period. The best-corrected visual acuity (BCVA) and anatomical findings were evaluated before the pandemic, during the pandemic, and at 6-, 12-, 18-, and 24-months post-pandemic. Results: The delayed and on-time groups comprised 54 and 72 patients, respectively. After the pandemic, the injection interval increased by 0.65 ± 1.51 months (*p* = 0.003), with 22.2% of the patients in the delayed group switching to the treat-and-extended regimen (*p* < 0.001). The delayed group showed greater mean BCVA deterioration (*p* = 0.027) and central subfield thickness (*p* = 0.037) at 6 months and worse maximum subretinal fluid height (*p* = 0.022) at 18 months than the on-time group. No difference was observed between the groups in the second year. Conclusion: the negative effects of delaying anti-VEGF treatment because of the COVID-19 pandemic can be ameliorated by changing the treatment regimen and shortening treatment intervals.

## 1. Introduction

Age-related macular degeneration (AMD) is a degenerative disease affecting the human retina, primarily manifesting in the macula lutea. This progressive degenerative disease leads to an irreversible loss of central vision in late-onset stages, significantly impacting the overall quality of life for patients [1,2]. The global prevalence of advanced age-related macular degeneration (AMD) is approximately 1–3% [1]. AMD is the leading cause of blindness in developed nations, accounting for ~8.7% of all cases of blindness worldwide. AMD is classified into dry and wet AMD based on the presence of choroidal neovascularization (CNV). Wet AMD, also referred to as neovascular AMD (nAMD), is characterized by the existence of CNV. Exudation or bleeding from this CNV results in various symptoms, such as subretinal fluid (SRF), pigment epithelial detachment, and subretinal hemorrhage, leading to deterioration in vision [2]. nAMD is responsible for almost 90% of cases of blindness associated with AMD.

Intravitreal anti-vascular endothelial growth factor (VEGF) therapy targets several key pathogenic pathways in nAMD [3] and plays an important role in preventing the progression and improving the prognosis of patients with nAMD [4,5]. There are two main regimens for anti-VEGF therapy, including the “pro re nata” (PRN) method, where injections are given as needed, and the “treat and extend” (T&E) method, where injections are given upon the detection of recurrence and are subsequently given at varying intervals in response to disease progression [6]. The visual acuity of patients with delayed, discontinuous, or irregular treatments tends to be worse than that of regularly treated patients, and their condition fails to improve even after frequent treatment and monitoring [7,8].

The coronavirus disease 2019 (COVID-19) pandemic has exerted an overwhelming burden on healthcare systems [9,10], with one of the consequences being that non-urgent outpatients avoided attending medical institutions for several months following the outbreak [11]. Therefore, timely treatment for patients with chronic ophthalmic conditions, such as diabetic retinopathy or AMD, has been delayed [9,12,13,14]. In particular, patients with nAMD are mostly geriatric patients at risk of developing severe COVID-19. Accordingly, to avoid infection, they delayed hospital visits and intravitreal anti-VEGF injections [15]. Therefore, the adverse effects of delayed treatment, such as visual impairment or retinal structural damage, need to be clarified. Moreover, tracking the long-term treatment progress of patients whose condition worsened due to delayed treatment is essential. This involves verifying clinical outcomes and understanding how their progress varies based on post-pandemic treatment methods. Identifying risk and preventive factors for the course is crucial for establishing treatment strategies for patients who may experience delayed treatment in similar pandemic situations in the future.

Several studies have followed patients who experienced delayed treatment because of the pandemic for up to one year [16,17]. In these studies, it was reported that delayed treatment due to COVID-19 in nAMD patients resulted in impaired BCVA, which did not recover over the subsequent year. However, there is a gap in these previous studies as they did not consider the impact of natural disease progression on nAMD [18,19], given that they did not compare it with a control group receiving timely treatment. In fact, although BCVA did not recover in a previous study, anatomic deterioration was reported to be fully restored [16]. Moreover, no study has applied a follow-up period of more than 2 years. Therefore, we assumed that there would be no significant difference in outcomes between patients who experienced delayed treatment and those who were treated promptly when observed over an extended period. We aimed to compare the outcomes of delayed and on-time intravitreal anti-VEGF treatment during the pandemic in patients with nAMD within a 2-year follow-up period. Our findings could inform the response of medical institutions to future pandemics, other emergency response scenarios, or treatment-limiting situations.

## 2. Materials and Methods

### 2.1. Ethical Consideration

This retrospective case–control study was approved by the Keimyung University Dongsan Hospital Institutional Review Board (approval number: 2022-11-010) and adhered to the principles of the Declaration of Helsinki and all the applicable guidelines for conducting research involving human subjects. The requirement for informed consent was waived, given the retrospective nature of this study, by the Keimyung University Dongsan Hospital Institutional Review Board on 11 November 2022 (approval number: DSMC 2022-11-010).

### 2.2. Study Design

**Figure 1:**
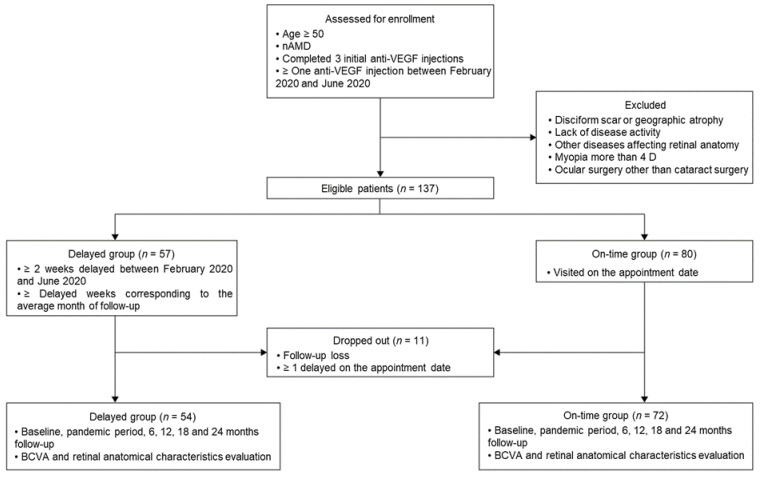
Flow diagram illustrating the study enrolment process. nAMD, neovascular age-related macular degeneration; VEGF, vascular endothelial growth factor; D, diopters.

We enrolled patients aged ≥ 50 years who had undergone intravitreal anti-VEGF therapy between February and June 2020. Furthermore, these patients had received 3-monthly intravitreal anti-VEGF injections before the pandemic period. The delayed group comprised patients whose anti-VEGF injections were postponed for ≥2 weeks from their scheduled date during the pandemic. The on-time group comprised patients who were promptly treated with injections during the same period. We excluded patients who were lost to follow-up for up to 2 years or who visited later than the scheduled appointment at least once after the pandemic period. We also excluded patients who did not require anti-VEGF injections because of a disciform scar, geographic atrophy, or lack of disease activity. Finally, we excluded patients with other diseases that may affect the retinal anatomical structure, including diabetic retinopathy, retinal vein occlusion, glaucoma, and myopia more than 4D, as well as those who underwent intraocular surgery other than cataract surgery (Supplementary Table S1). To eliminate the confounding effect of the similarity of measures within the same person, we only selected the most recently treated eye for the analysis (Figure 1).

### 2.3. Clinical Data Collection

We analyzed each patient’s medical records at baseline (during their last visit before the pandemic), during the pandemic, and 6-, 12-, 18-, and 24- months after the pandemic. Data from the date of their first injection were utilized for patients who received multiple injections during the pandemic period. Best-corrected visual acuity (BCVA) was recorded using the logarithm of the minimum angle of resolution (logMAR) scale. The demographic characteristics included age, sex, number of injections before baseline, period from the initial diagnosis to first injection during the pandemic, and duration of delay after the original appointment date. We typically treat patients with nAMD with 3-monthly loading injections, followed by treatment using the PRN method. In case of deterioration or recurrence, the treatment method was changed to the T&E regimen [20]. If the last eye was affected or both eyes had nAMD, the T&E regimen could be started immediately after the monthly loading injections. The treatment protocols were documented as the T&E or PRN regimen. Moreover, the injection intervals for each group were recorded.

### 2.4. Anatomical Data Collection

During the patient visits, swept-source optical coherent tomography was conducted using a DRI OCT Triton Plus (Topcon Co., Tokyo, Japan, Catalog No.: Triton). Using the obtained images, we analyzed the central subfield thickness (CST) and the presence/absence of SRF, subretinal hyper-reflective material (SHRM), and intraretinal fluid (IRF) in a 6 × 6 mm region around the fovea. We utilized the built-in IMAGEnet 6 capture software (IMAGEnet 6 version 1.28, Topcon Co., Tokyo, Japan) to automatically measure the average thickness of the retina within a 1 mm radius from the center of the fovea, corresponding to the CST. The presence of SRF was identified by observing a consistent dark area (fluid) between the outer retina and the hyper-reflective line of the retinal pigment epithelium (RPE) in the OCT images. SHRM was recognized as a morphological feature visible on the OCT images, appearing as hyper-reflective material situated externally to the retina and internally to the RPE. IRF presence was confirmed when it manifested as dark cystic accumulations of fluid above the outer plexiform layer of the retina.

**Figure 2:**
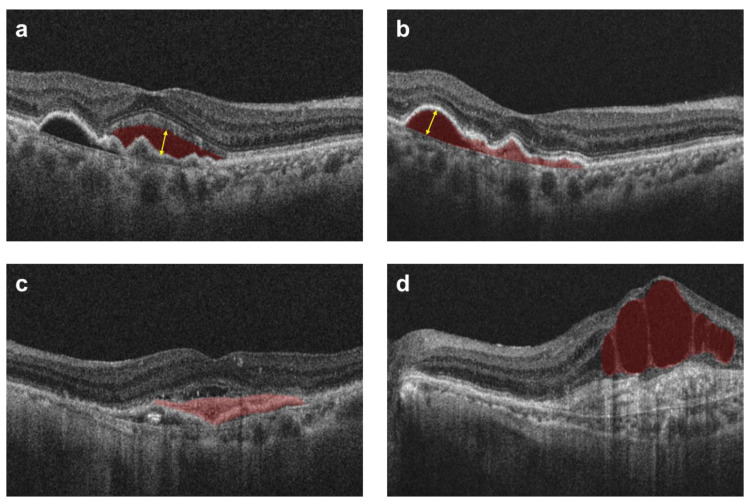
Acquisition of anatomical data. The presence of SRF can be identified in a dark fluid area above the hyper-reflective line of the RPE (Red area in panel (**a**)), while PED can be identified below the RPE line, presenting as either a dark fluid or bright vascular area (Red area in panel (**b**)). The measurement of the (**a**) maximum SRF height involved assessing the maximum distance between the boundary of the outer retina and the RPE (Yellow arrow in panel (**a**)), whereas the (**b**) maximum PED height was determined as the maximum distance between the RPE and the inner surface of the Bruch membrane (Yellow arrow in panel (**b**)). (**c**) SHRM was confirmed by the presence of a hyper-reflective cluster between the retina and RPE (Red area in panel (**c**)), and (**d**) the presence of IRF was assessed through the existence of intraretinal cystic dark fluid areas (Red area in panel (**d**)). SRF, subretinal fluid; PED, pigment epithelial detachment; RPE, retinal pigment epithelium; SHRM, subretinal hyper-reflective material; IRF, intraretinal fluid.

Moreover, the maximum height of both SRF and pigment epithelial detachment (PED) measurements were taken in micrometers. The maximum SRF height was determined as the maximum distance between the outer retina and the RPE line in the OCT images, utilizing the caliper tool in the IMAGEnet 6 software. PED presence was established when a dark fluid area was observed beneath the RPE. The maximum PED height was measured as the greatest distance between the inner surface of the Bruch membrane and the outer surface of the RPE (Figure 2). A single researcher (J.-G.K) reviewed and documented all the images and observations.

### 2.5. Statistical Analysis

The sample size was calculated using G*Power 3.1.9.2 software (Franz Faul at the University of Kiel, Kiel, Germany) [21]. To detect a difference between two independent groups in a repeated measures analysis of variance (RM ANOVA) with 95% power, a *p*-value of 0.05, and an effect size of 0.25, a total sample size of 124 participants was required. Given that the sample sizes of both groups exceeded 30, the quantitative variables were considered to be normally distributed. Descriptive statistics were used to analyze the baseline characteristics. Moreover, between-group comparisons were performed using the independent *t*-test and chi-square test. Between-period changes in the injection interval and differences in the treatment regimens were analyzed using the paired *t*-test and McNemar’s test, respectively. An independent *t*-test was used for between-group comparisons of the baseline BCVA, CST, maximum SRF height, and PED. Changes from baseline to each time point were compared using a two-way RM ANOVA. Between-group comparisons of the proportions of SRF, SHRM, and IRF at each time point were performed using the chi-square test. A multiple linear regression analysis was performed to investigate the factors related to changes in BCVA, CST, and the maximum height of the SRF throughout the study period. The Mann–Whitney U test was performed to assess the between-regimen differences in injection intervals after the pandemic. Finally, between-sex differences in the deterioration of BCVA, CST, and the maximum height of the SRF were compared using a two-way RM ANOVA. Statistical analyses were performed using IBM SPSS Statistics version 25.0.0 (IBM Co., Armonk, NY, USA). Statistical significance was defined as a two-tailed *p*-value of ≤0.05.

## 3. Results

### 3.1. Baseline Characteristics

Among the 126 included patients (126 eyes), 54 and 72 patients were assigned to the delayed and on-time groups, respectively, as shown in Figure 1. The average ages in the delayed and on-time groups were 73.5 ± 6.5 (58–85) and 74.0 ± 6.7 (58–87) years, respectively. There were 15 (27.8%) and 27 (37.5%) women in the delayed and on-time groups, respectively. Among the patients in the delayed group, 34 patients had occult choroidal neovascularization (CNV), 17 patients had classic CNV, and 3 patients had retinal angiomatous proliferation. In the on-time group, there were 47, 18, and 7 patients with each respective condition. Additionally, there were 18 and 34 left eyes affected in the delayed and on-time groups, respectively. Before the pandemic, the delayed and on-time groups received 14.3 ± 10.0 (3–51) and 13.5 ± 8.5 (3–49) injections, respectively. The interval from the diagnosis to the pandemic was 46.65 ± 32.71 (6–164) and 44.26 ± 32.29 (4–124) months in the delayed and on-time groups, respectively. There were no significant between-group differences in the baseline characteristics. In the delayed group, the mean duration from the appointment date to treatment was 1.48 ± 1.28 (from 0.4 to 9) months (Table 1). All patients had the same ethnic background (Korean).

### 3.2. Changes in Injection Intervals

The pre-pandemic injection intervals in the delayed and on-time groups were 3.06 ± 1.48 (1.0–8.6) and 2.80 ± 1.48 (1.0–12.0) months, respectively. There were no significant between-group differences in the injection interval (*p* = 0.326). In the delayed group, the post-pandemic injection intervals decreased by 0.65 ± 1.52 months (*p* = 0.003) (Table 2).

### 3.3. Changes in Treatment Regimens

The T&E regimen was administered to 35 (64.8%) and 57 (79.2%) patients in the delayed and on-time groups, respectively. There were no significant between-group differences in the treatment regimen (*p* = 0.104). In the delayed group, 12 (22.2%) patients were switched to the T&E regimen after the pandemic (*p* < 0.001). However, this trend of a change in regimen was not observed in the on-time group (Table 2).

### 3.4. Comparison of BCVA and OCT Measurements

**Figure 3:**
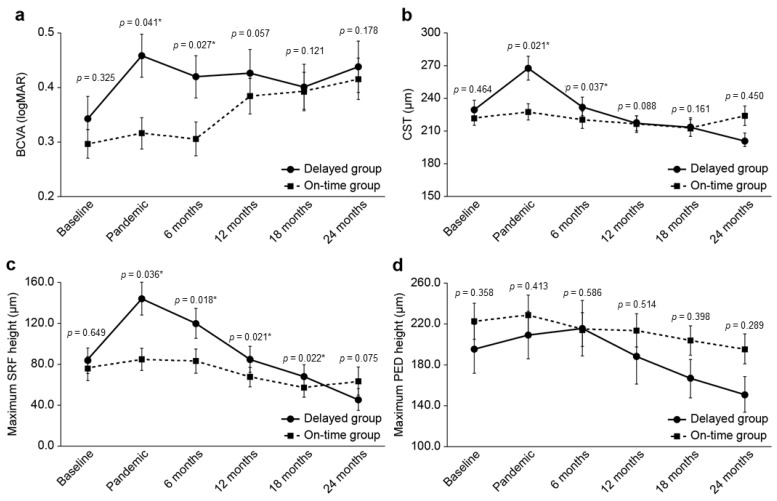
Line graphs illustrating the deterioration of (**a**) BCVA and (**b**) CST and (**c**) the maximum SRF height in the delayed group following the pandemic, with eventual recovery in the second year. (**d**) No differences were observed in the maximum height of PED between the groups during the study period. BCVA, best-corrected visual acuity; CST, central subfield thickness; SRF, subretinal fluid; PED, pigment epithelial detachment. *: statistically significant (between groups).

There were no significant between-group differences in the baseline BCVA, CST, maximum SRF, or PED height (*p* = 0.325, 0.464, 0.649, and 0.358, respectively). The BCVA, CST, and maximum SRF height showed greater deterioration in the delayed group than in the on-time group at 6 months post-pandemic (*p* = 0.027, 0.037, and 0.018, respectively). Furthermore, the maximum SRF height showed greater deterioration in the delayed group than in the on-time group for up to 18 months post-pandemic (*p* = 0.022). At 6 months, the mean BCVA and CST were higher by 0.10 ± 0.05 logMAR and 19.9 ± 9.4 µm, respectively, in the delayed group compared to the on-time group. At 12 months post-pandemic, there were no significant between-group differences in the BCVA and CST (*p* = 0.057 and 0.088, respectively). At 18 months post-pandemic, the mean maximum SRF height was higher in the delayed group than in the on-time group by 26.5 ± 11.4 µm; however, there was no significant between-group difference at 24 months post-pandemic (Table 3). Figure 3 shows the between-group comparisons of the progression of each parameter.

### 3.5. Comparisons of Anatomical Findings

**Figure 4:**
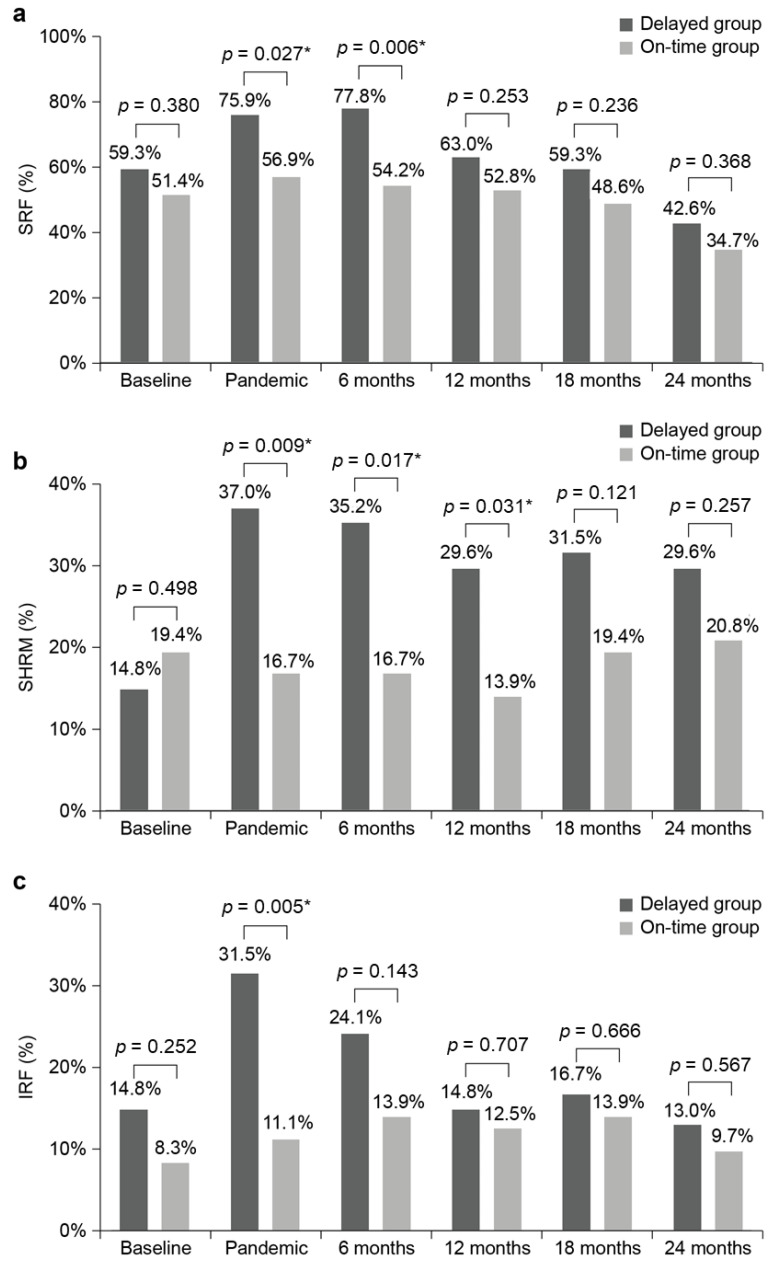
Bar graphs depicting the prevalence of (**a**) SRF, (**b**) SHRM, and (**c**) IRF, illustrating an increase followed by subsequent recovery in the delayed group throughout the study period. SRF, subretinal fluid; SHRM, subretinal hyper-reflective material; IRF, intraretinal fluid. *: statistically significant (between groups).

There were no significant between-group differences in the baseline proportions of SRF, SHRM, and IRF (*p* = 0.380, 0.498, and 0.252, respectively). The delayed group had more SRF, SHRM, and IRF during the pandemic than the on-time group (*p* = 0.027, 0.009, and 0.005, respectively). Specifically, SRF was more frequently detected in the delayed group at 6 (*p* = 0.006) and 12 (*p* = 0.031) months post-pandemic than in the on-time group; however, there were no significant differences at subsequent follow-up time points. There were no between-group differences in the proportions of SRF, SHRM, and IRF after 18 months post-pandemic (*p* = 0.236, 0.121, and 0.666, respectively) (Table 4). Figure 4 shows the between-group comparisons of the anatomical findings at each time point.

### 3.6. Risk Factors Associated with Disease Progression

Age, previous anti-VEGF injections, the pre-pandemic injection interval, and the interval from diagnosis to the pandemic were not significantly correlated with changes in the BCVA, CST, or maximum SRF height. The CST and maximum SRF height showed greater improvements in men than in women (standardized beta coefficient = 0.217 and 0.318, *p* = 0.217 and 0.001, respectively). The post-pandemic injection interval was negatively correlated with improvements in CST and maximum SRF height (standardized beta coefficient = −0.292 and −0.298, *p* = 0.009 and 0.005, respectively) (Supplementary Table S2).

### 3.7. Worsening of Clinical Signs Based on Sex

Given these findings, we assumed that sex-based differences would be evident in the deterioration during the pandemic. There were no significant between-sex differences in the post-pandemic deterioration of BCVA (*p* = 0.900); however, men exhibited greater deterioration in CST and maximum SRF height than women (*p* = 0.005 and 0.017, respectively) (Supplementary Table S3).

## 4. Discussion

We previously showed that delayed anti-VEGF injections due to the COVID-19 pandemic significantly aggravated BCVA and anatomical pathology, and the condition did not improve to the pre-pandemic status 6 months after the onset of the pandemic [22]. However, our previous study was limited by the lack of a control group and the short follow-up period (6 months). The present findings showed that reducing treatment intervals and switching to the T&E method during the pandemic ameliorated the negative effects of treatment delays for a certain period (Figure 3), including the occurrence of anatomical lesions (Figure 4).

The BCVA and anatomical findings of the patients did not return to the pre-pandemic status even after 1 post-pandemic year, which is consistent with the results of previous studies [16,17]. However, these results should be interpreted with caution because patients with nAMD may exhibit a worsening long-term course despite receiving appropriate treatment during the follow-up period [18,19]. In the present study, both BCVA and CST showed no recovery within 1 year post-pandemic in the delayed group. However, the on-time group also showed BCVA deterioration at 1 year post-pandemic, necessitating the exclusion of the effect of the natural disease course. To minimize this effect, we used a control group. No significant between-group differences in the deterioration of BCVA or CST were observed at 1 year.

The maximum SRF height showed a relatively worse course in the delayed group until the 18th follow-up month compared to the other parameters. As previously mentioned [22], the slow recovery of SRF in the delayed group could be attributed to the inclusion of patients with nAMD and refractory SRF [23,24]. In such patients, the SRF showed a relative response to intravitreal anti-VEGF injections even after changing the treatment regimen and shortening the injection interval. However, these patients can also show long-term improvement with steady short-interval injection treatments [25].

In patients with nAMD, delayed treatment can lead to the long-term deterioration of clinical findings [8,26]. However, we found no significant between-group differences in the BCVA and anatomical parameters after long-term treatment. Therefore, we investigated the factors influencing rapid recovery. We found that sex and the post-pandemic injection interval were significantly associated with changes in the CST and maximum height of SRF during the 24-month follow-up period (Supplementary Table S2).

Men showed greater improvement in CST and maximum SRF height than women. Sex differences in AMD progression remain unclear, with some studies reporting faster AMD progression in women under the influence of estrogen reduction [27,28] and others reporting no sex-related differences [29,30]. In the present study, both the CST and maximum SRF height worsened from the baseline to the pandemic onset to a greater extent in men than in women, and this finding may explain the greater improvement observed in men after 2 years post-pandemic. Notably, men received a significantly higher number of previous anti-VEGF injections than women, and this could indicate a longer disease course, poorer treatment response, and greater vitreomacular interface changes in women [31,32]. The postponement of treatment may have led to greater anatomical deterioration in women [33,34].

Furthermore, the post-pandemic injection interval was negatively correlated with improvements in the CST and maximum SRF height. The post-pandemic injection interval was significantly shorter in the delayed group than in the on-time group. Therefore, frequent and intensive injections after the pandemic helped to gradually alleviate the exacerbated anatomical changes in the delayed group [35,36], and this finding could explain the lack of between-group differences in the 2-year prognosis. In the delayed group, there was a significant increase in the proportion of patients who switched to the T&E regimen after the pandemic. Among patients with exacerbated nAMD, maintaining a PRN regimen is associated with greater visual deterioration than switching to a T&E regimen [37,38]. In the present study, patients who switched to the T&E regimen had a significantly shorter injection interval than those who maintained PRN treatment. Taken together, changing the treatment regimen and shortening the injection interval improved the anatomical parameters that had deteriorated during the pandemic, and this approach contributed to the absence of between-group differences in the 2-year prognosis.

The strength of this study is that it established a control group to determine the sole effect of delayed injections and eliminate the influence of disease progression. Moreover, it involved a long-term follow-up period of up to 2 years. However, this study has several limitations. First, this was a retrospective study; therefore, the presence of selection bias cannot be ignored. Second, only 54 patients remained in the delayed group at the 2-year follow-up point. Considering the study design, a larger sample size would have yielded more robust results. Third, given that this study was conducted in a tertiary medical center, it may be challenging to generalize the findings to the general population. Fourth, we did not identify factors affecting the changes in BCVA. We found no between-group difference in the change in BCVA from the first year to after the pandemic; therefore, future studies should focus on identifying these factors. Finally, studies with longer follow-up periods are needed because the disease course may change after 2 years.

In conclusion, patients with delayed intravitreal anti-VEGF injections due to COVID-19 exhibited temporary deterioration in both BCVA and anatomical signs. However, when observed up to the second year, there was no significant difference compared to patients who received timely treatment. This recovery appeared to be associated with switching to a T&E regimen and a reduction in injection intervals. Therefore, in the event of a similar global crisis that delays the treatment of patients with nAMD, the long-term effect of delayed injections can be minimized through proactive and consistent treatment plans.

## Figures and Tables

**Table 1:** No differences in the baseline demographics and characteristics between the groups.

Characteristics	Delayed Group	On-Time Group	*p*-Value
Number of eyes included (***n***)	54	72	-
Age (***years***)	73.5 ± 6.5	74.0 ± 6.7	0.684 ^a^
Sex (men:women)	39:15	45:27	0.340 ^b^
Type of CNV (occult:classic:RAP)	34:17:3	47:18:7	0.558 ^b^
Laterality (right:left)	36:18	38:34	0.144 ^b^
Previous anti-VEGF injection (***times***)	14.3 ± 10.0	13.5 ± 8.5	0.634 ^a^
Period from diagnosis to pandemic injection (***months***)	46.65 ± 32.71	44.26 ± 32.29	0.813 ^a^
Delayed length of time (***months***)	1.48 ± 1.28	-	-

Data are reported either as means with standard deviations or as numerical values. CNV: choroidal neovascularization; RAP: retinal angiomatous proliferation; VEGF: vascular endothelial growth factor. ^a^: independent *t*-test; ^b^: chi-square test.

**Table 2:** Shifting to more proactive treatments in the delayed group following the COVID-19 pandemic.

		Pre-Pandemic	Post-Pandemic	*p*-Value
**Injection interval (*months*)**	Delayed group	3.06 ± 1.48	2.41 ± 1.47	0.003 ^a^*
	On-time group	2.80 ± 1.48	2.75 ± 1.35	0.662 ^a^
**Treatment regimen (PRN:T&E)**	Delayed group	19:35	7:47	<0.001 ^b^*
	On-time group	15:57	10:62	0.180 ^b^

Data are reported either as means with standard deviations or as numerical values. COVID, coronavirus disease; PRN, pro re nata; T&E, treat-and-extend. ^a^: paired *t*-test, ^b^: McNemar’s test, *: statistically significant.

**Table 3:** Transient deterioration and subsequent recovery of BCVA and OCT measurements in the delayed group.

		Baseline	Pandemic	6 Months	12 Months	18 Months	24 Months
**BCVA (*logMAR*)**	Delayed group	0.34 ± 0.30	0.46 ± 0.29 *	0.42 ± 0.28 *	0.43 ± 0.32	0.40 ± 0.30	0.44 ± 0.35
	On-time group	0.30 ± 0.22	0.32 ± 0.24 *	0.31 ± 0.26 *	0.38 ± 0.28	0.39 ± 0.30	0.42 ± 0.32
**CST (** ***µm*** **)**	Delayed group	229.3 ± 63.7	267.3 ± 78.5 *	232.1 ± 61.8 *	216.9 ± 49.9	213.4 ± 63.4	201.4 ± 45.9
	On-time group	221.5 ± 54.4	227.4 ± 64.5 *	220.0 ± 66.6 *	216.3 ± 65.0	212.5 ± 66.0	223.6 ± 76.0
**Maximum SRF height (** ***µm*** **)**	Delayed group	83.2 ± 89.8	143.6 ± 115.2 *	119.7 ± 103.7 *	85.0 ± 91.0 *	67.9 ± 85.4 *	45.3 ± 76.3
	On-time group	75.4 ± 87.3	84.5 ± 82.8 *	82.9 ± 88.9 *	67.3 ± 73.1 *	57.0 ± 69.8 *	63.3 ± 103.7
**Maximum PED height (** ***µm*** **)**	Delayed group	195.2 ± 172.3	208.6 ± 165.0	215.2 ± 193.9	187.8 ± 191.6	166.4 ± 136.0	151.2 ± 124.8
	On-time group	222.0 ± 149.6	228.2 ± 165.4	213.9 ± 140.0	213.1 ± 138.0	203.2 ± 120.6	195.2 ± 123.6

Data are reported either as means with standard deviations or as numerical values. BCVA, best-corrected visual acuity; CST, central subfield thickness; SRF, subretinal fluid; PED, pigment epithelial detachment. *: statistically significant (between groups).

**Table 4:** More anatomical findings were observed in the delayed group during the pandemic and within a year after the pandemic, with no significant differences noted after 18 months.

		Baseline	Pandemic	6 Months	12 Months	18 Months	24 Months
**SRF, *n* (*%*)**	Delayed group	32 (59.3)	41 (75.9) *	42 (77.8) *	34 (63.0)	32 (59.3)	23 (42.6)
	On-time group	37 (51.4)	41 (56.9) *	39 (54.2) *	38 (52.8)	35 (48.6)	25 (34.7)
**SHRM, *n* (*%*)**	Delayed group	8 (14.8)	20 (37.0) *	19 (35.2) *	16 (29.6) *	17 (31.5)	16 (29.6)
	On-time group	14 (19.4)	12 (16.7) *	12 (16.7) *	10 (13.9) *	14 (19.4)	15 (20.8)
**IRF, *n* (*%*)**	Delayed group	8 (14.8)	17 (31.5) *	13 (24.1)	8 (14.8)	9 (16.7)	7 (13.0)
	On-time group	6 (8.3)	8 (11.1) *	10 (13.9)	9 (12.5)	10 (13.9)	7 (9.7)

Data are reported as numerical values with percentages. SRF, subretinal fluid; SHRM, subretinal hyper-reflective material; IRF, intraretinal fluid. *: statistically significant (between groups).

## Data Availability

The datasets generated during and/or analyzed during the current study are available from the corresponding author on reasonable request. K.T.K. had full access to all the data in the study and takes responsibility for the integrity of the data and accuracy of the data analysis.

## Supplementary Materials

The following supporting information can be downloaded at: https://www.mdpi.com/article/10.3390/jcm13030867/s1, Table S1: Inclusion and exclusion criteria; Table S2: A longer post-pandemic injection interval and being male were the risk factors for worsened BCVA and OCT parameters at 2 years post-pandemic; Table S3: Men exhibited worsened OCT parameters compared to women during the pandemic period.
