# Supplementary material for: Two-Year Follow-Up Study of Patients with Neovascular Age-Related Macular Degeneration Undergoing Anti-VEGF Treatment during the COVID-19 Pandemic
Source: J Clin Med. 2024 Feb 1;13(3):867. doi: 10.3390/jcm13030867 (PMC10856664; doi:10.3390/jcm13030867)
Supplement: Supplementary file 1 [file jcm-13-00867-s001.zip › jcm-2830684-supplementary.pdf]

Table S1. Inclusion and exclusion criteria

Inclusion criteria	Exclusion criteria
nAMD patient aged ≥ 50 years	AMD with a disciform scar or geographic atrophy
Completed three initial monthly intravitreal anti-VEGF injections	Did not need intravitreal anti-VEGF injection due to lack of disease activity
At least one delayed intravitreal anti-VEGF injection between February and June 2020	Higher myopia more than 4 D
≥2 weeks delayed for scheduled injection date between February 2020 and June 2020 (Delayed group)	Diseases that could affect anatomical structure of the retina (e.g. diabetic retinopathy, retinal vein occlusion, etc.)
Delayed more than weeks corresponding to the average month of follow-up (Delayed group)	History of intraocular surgery except for cataract surgery
Visited on the appointment date (On-time group)	Follow-up loss or delayed during study period

nAMD, neovacular age-related macular degeneration; VEGF, vascular endothelial growth factor; D, diopters.

Table S2. Longer post-pandemic injection interval and being men were the risk factors for worsened BCVA and OCT parameters at 2 years post-pandemic

	**BCVA**		**CST**		**Maximum SRF height**	
	S.E.	*p*-Value	S.E.	*p*-Value	S.E.	*p*-Value
Age (*years*)	0.000	1.000	0.009	0.917	−0.043	0.607
Sex (in men, compared to in women)	0.139	0.138	0.217	0.016*	0.318	<0.001*
Previous anti-VEGF injection (*times*)	0.019	0.869	0.093	0.396	0.016	0.876
Injection interval before the pandemic (*months*)	−0.004	0.974	0.177	0.141	0.053	0.644
Injection interval after the pandemic (*months*)	0.039	0.736	−0.292	0.009*	−0.298	0.005*
Interval from diagnosis to the pandemic (*months*)	−0.093	0.414	−0.059	0.588	−0.069	0.502

BCVA, best-corrected visual acuity; CST, central subfield thickness; SRF, subretinal fluid; VEGF, vascular endothelial growth factor; S.E., standardized beta coefficient
* Statistically significant.

Table S3. Men exhibited worsened OCT parameters compared to women during the pandemic period

		Baseline	Pandemic	*p*-Value
BCVA (*logMAR*)	Men	0.31±0.22	0.39±0.25	0.900
	Women	0.33±0.32	0.36±0.32	
CST (μm)	Men	232.0±63.0	257.5±76.8	0.005*
	Women	210.5±45.4	218.5±58.1	
Maximum SRF height (μm)	Men	86.0±90.4	130.9±105.5	0.017*
	Women	63.3±81.8	71.0±86.3	

Data are reported either as means with standard deviations or as numerical values. BCVA, best-corrected visual acuity; CST, central subfield thickness; SRF, subretinal fluid.

* Statistically significant (between men and women).
